# MiR-24-3p Inhibits the Progression of Pancreatic Ductal Adenocarcinoma Through LAMB3 Downregulation

**DOI:** 10.3389/fonc.2019.01499

**Published:** 2020-01-21

**Authors:** Wenjie Huang, Jianyou Gu, Tian Tao, Junfeng Zhang, Huaizhi Wang, Yingfang Fan

**Affiliations:** ^1^Department of First Hepatobiliary Surgery, Zhujiang Hospital, Southern Medical University, Guangzhou, China; ^2^Institute of Hepatopancreatobiliary Surgery, Chongqing General Hospital, University of Chinese Academy of Sciences, Chongqing, China; ^3^Institute of Hepatopancreatobiliary Surgery, Southwest Hospital, Army Medical University, Chongqing, China

**Keywords:** miR-24-3p, LAMB3, pancreatic ductal adenocarcinoma (PDAC), proliferation, invasion

## Abstract

Pancreatic ductal adenocarcinoma (PDAC) is associated with several genetic syndromes. However, the molecular mechanism underlying PDAC progression is still unknown. In this study, we showed that Laminin Subunit Beta 3 (LAMB3) was aberrantly overexpressed in PDAC and was closely associated with the overall survival rate of patients with PDAC. Functional studies demonstrated that LAMB3 played important roles in cell proliferation, the cell cycle, and invasion capacity. Using bioinformatics analysis, we determined that miR-24-3p was an upstream miRNA of LAMB3, and further experiments verified that miR-24-3p regulated LAMB3 expression in PDAC cells. A dual-luciferase reporter system demonstrated that miR-24-3p directly targeted the LAMB3 3'UTR, and FISH assay confirmed that miR-24-3p and LAMB3 mRNA mostly resided in cytoplasm, accounting for their post-translational regulation. Rescue assay demonstrated that miR-24-3p exerted its anti-cancer role by suppressing LAMB3 expression. Finally, by using a subcutaneous xenotransplanted tumor model, we demonstrated that miR-24-3p overexpression inhibited the proliferation of PDAC by suppressing LAMB3 expression *in vivo*. Collectively, our results provide evidence that the miR-24-3p/LAMB3 axis plays a vital role in the progression of PDAC and indicate that the miR-24-3p/LAMB3 axis may represent a novel therapeutic target for PDAC.

## Introduction

Pancreatic ductal adenocarcinoma (PDAC) is one of the deadliest malignancies, with a mortality rate that is almost identical to its morbidity rate. Based on the cancer mortality data provided by the National Center for Health Statistics (NCHS), the latest authoritative study reveals that PDAC will be one of the leading fatal cancer types, accounting for 7% of estimated new cancer deaths (1). A previous study has demonstrated that pancreatic cancer is a genetic disease accompanied by the activation of oncogenes and loss of tumor suppressor genes (2). Accumulation of gene mutations has been proven to play important roles in PDAC initiation, progression, metastasis, and chemoresistance (3–7). Considering the key roles of gene mutations in the evolutionary process of pancreatic cancer, novel target molecules are urgently needed to improve the early diagnosis, treatment, and prognosis of pancreatic cancer.

MiRNAs are a class of small non-coding regulatory RNAs that are ~21 nucleotides in length. The first miRNA was described ~20 years ago (8). In the past few decades, both the biogenesis and biological role of miRNAs have been extensively investigated (9). Previous studies have demonstrated that miRNAs play important roles in mammalian development and that their dysregulation results in a wide range of human diseases, including malignant cancers (10). Therefore, miRNAs are widely involved in several events in cancer progression such as proliferation, metastasis, chemoresistance, and epithelial-mesenchymal transition (EMT) (11–14). Depending on the target gene repressed, miRNAs can act as oncomiRs or tumor suppressors, and a certain miRNA can have controversial functions in different cancers (15–17). Not surprisingly, miRNAs are also involved in the development of PDAC (18), but their role is still unclear and remains to be investigated.

Laminin subunit beta 3 (LAMB3) belongs to the laminin family, one of the most common basement membrane protein families (19). Laminin is widely involved in many biological processes such as attachment, migration, and interaction with other extracellular matrix components. It is widely recognized that LAMB3 also plays important roles in the tumorigenesis and progression of thyroid cancer, papillary thyroid cancer, hepatocellular carcinoma, and pancreatic cancer (20–22). However, the underlying regulatory mechanism of LAMB3 in PDAC is still unclear.

In this study, we demonstrated that LAMB3 was upregulated in PDAC and served as a negative prognostic factor. Using bioinformatics analysis, we found that miR-24-3p was an upstream miRNA of LAMB3. Mechanistic investigations validate the potential interaction between miR-24-3p and LAMB3. In conclusion, our results indicate that the miR-24-3p/LAMB3 axis plays vital roles in the cell proliferation, cell cycle, and invasion capacity of PDAC *in vitro* and *in vivo* and that targeting the miR-24-3p/LAMB3 axis may represent a novel therapeutic strategy for PDAC.

## Results

### LAMB3 Is Upregulated in PDAC and Correlates With Poor Prognosis

**Figure 1:**
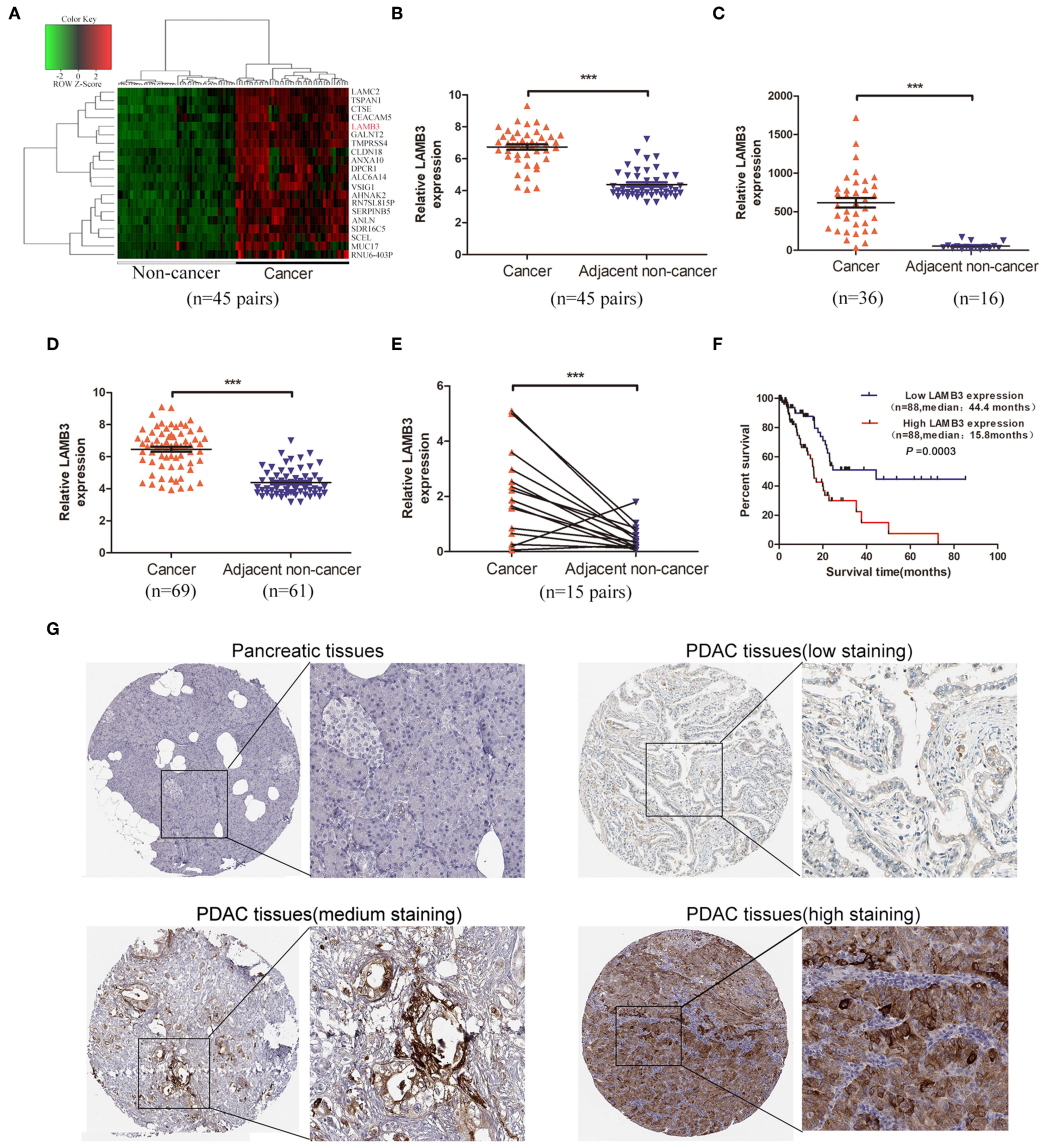
LAMB3 is upregulated in PDAC tissues and is associated with PDAC prognosis. **(A)** The cluster heat map showed that LAMB3 was overexpressed in PDAC tissues. **(B)** GEO GSE28375 dataset, **(C)** GSE16515 dataset, and **(D)** GSE62452 dataset confirmed that LAMB3 was overexpressed in PDAC tissues. **(E)** Higher expression levels of LAMB3 were detected using qRT-PCR in 15 matched PDAC tissues. Data are presented as mean ± SD; paired or unpaired *t*-test; ***p < 0.001. **(F)** Kaplan-Meier analysis revealed that LAMB3 overexpression was associated with poorer overall survival of PDAC patients. *p* < 0.001, log-rank test. **(G)** IHC (immunohistochemistry) results from the Human Protein Atlas demonstrated that LAMB3 was upregulated in PDAC tissues.

To our knowledge, LAMB3 is upregulated in a wide range of tumor types, but its expression profile in PDAC is still unclear. To explore the expression level of LAMB3 in PDAC, we first analyzed the GEO GSE28735 dataset, exploring the mRNA expression profile of 45 matched PDAC tumors and adjacent non-tumor tissues. The cluster heat map shows the top 20 upregulated mRNAs (Figure 1A). Further analysis revealed that LAMB3 was significantly upregulated in PDAC tissues (Figure 1B). The GEO datasets GSE16515 (Figure 1C) and GES62452 (Figure 1D) also validated this result. We examined LAMB3 expression in 15 paired PDAC tissues using qRT-PCR. The results consistently demonstrated that LAMB3 was overexpressed in PDAC tissues (Figure 1E). To determine whether the expression of LAMB3 correlated with the overall survival rates of PDAC, we further analyzed the clinical data of 176 pancreatic cancer patients from The Cancer Genome Atlas (TCGA). Kaplan-Meier survival curve analysis showed that patients with high LAMB3 expression had lower survival rates compared to patients with low LAMB3 expression (44.4 vs. 15.8667 months, *p* < 0.001, Figure 1F). Immunohistochemistry (IHC) results from the Human Protein Atlas (http://www.proteinatlas.org) revealed that LAMB3 was upregulated in PDAC tissues but was nearly undetectable in normal pancreatic tissues and that it was mainly localized in the cytoplasm and membrane [Figure 1G; (23)]. Taken together, these results suggest that LAMB3 is overexpressed in pancreatic cancer tissues and may be used as a prognostic indicator.

### LAMB3 Is Responsible for the Cell Proliferation, Cell Cycle, and Invasion Ability of PDAC

**Figure 2:**
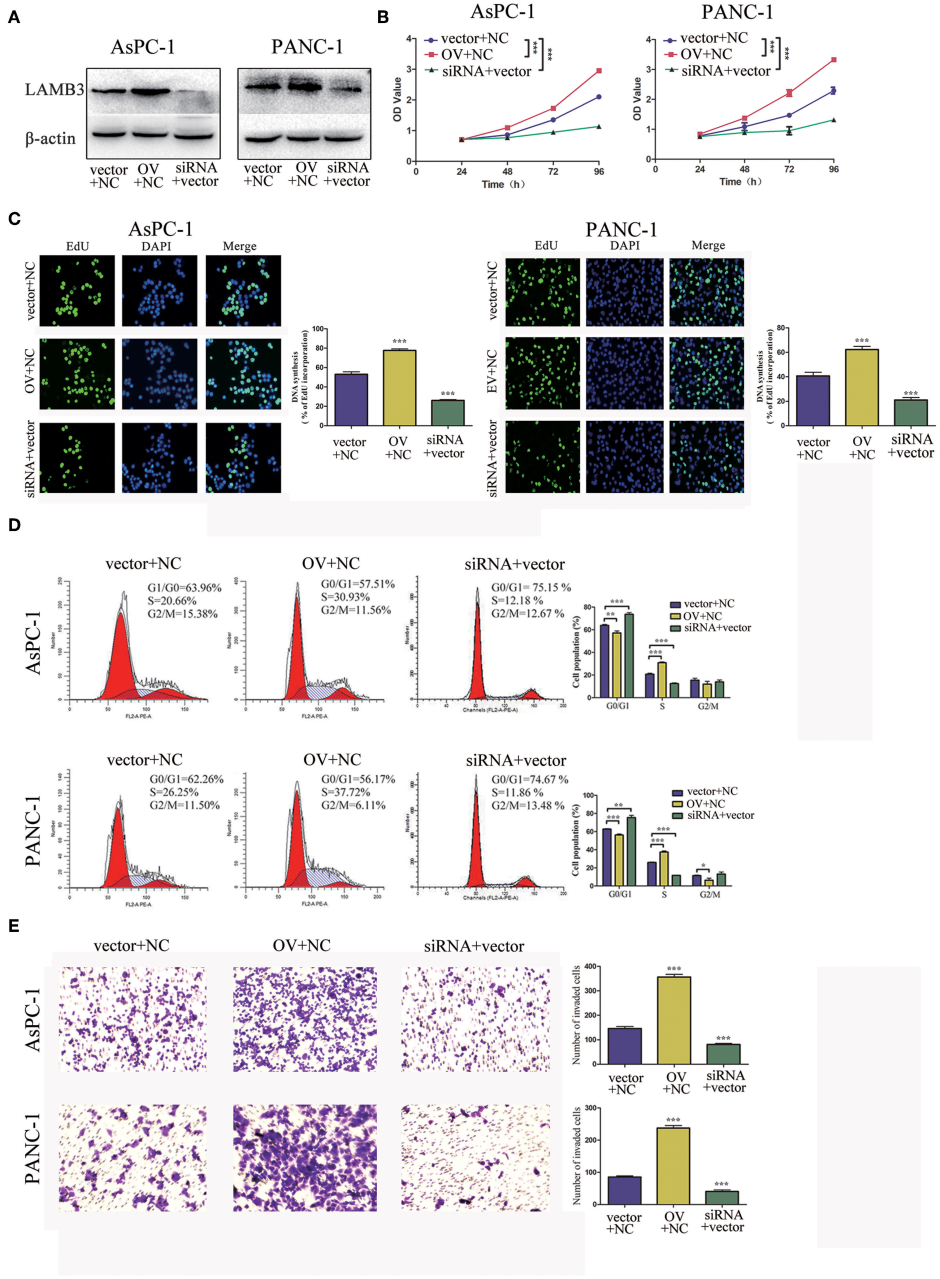
LAMB3 plays important roles in PDAC progression. **(A)** LAMB3 expression levels were determined using Western blotting analysis in cells transfected with LAMB3 overexpression vector (OV), LAMB3 siRNA. Cells transfected with LAMB3 empty vector (EV) and siRNA NC were used as the negative controls. **(B)** CCK-8 assays and **(C)** EdU assays confirmed that LAMB3 overexpression enhanced cell proliferation, whereas LAMB3 knockdown inhibited cell proliferation. **(D)** Flow cytometry showed that LAMB3 overexpression accelerated the cell cycle period and that LAMB3 knockdown induced cell cycle arrest. **(E)** A representative summary of the metastasis assay showed that LAMB3 overexpression promoted cell invasion, whereas LAMB3 knockdown inhibited cell invasion. Data are presented as mean ± SD of three independent experiments. *p < 0.05, **p < 0.01, ***p < 0.001, student's *t*-test.

We explored the biological roles of LAMB3 in PDAC progression *in vitro*. LAMB3 was knocked down by siRNA and was ectopically expressed using an overexpression vector (OV) in two PDAC cell lines, AsPC-1 and PANC-1. The siRNA NC and empty vector (EV) were co-transfected into AsPC-1 and PANC-1 cells as negative controls. Western blotting showed that LAMB3 expression in siRNA-transfected cells decreased, whereas it increased in OV-transfected cells (Figure 2A). CCK-8 and EdU incorporation assays were used to determine the role of LAMB3 in cell proliferation. The results showed that LAMB3 overexpression promoted cell proliferation, whereas its knockdown inhibited proliferation compared to the negative control (Figures 2B,C). As cell cycle dysregulation is an important event for cancer proliferation (24), we further investigated the function of LAMB3 in the cell cycle using flow cytometry analysis. The results demonstrated that LAMB3 overexpression accelerated the cell cycle, whereas low expression of LAMB3 induced cell cycle arrest (Figure 2D). At the same time, Transwell assays were performed to investigate whether LAMB3 affected the invasion ability of PDAC cells. The results revealed that LAMB3 overexpression enhanced cell invasion ability, whereas its knockdown induced the opposite effect (Figure 2E). In conclusion, LAMB3 is overexpressed in PDAC tissues and plays important roles in cell proliferation, the cell cycle, and the invasion capacity of PDAC cells.

### LAMB3 Is Directly Targeted by miR-24-3p

**Figure 3:**
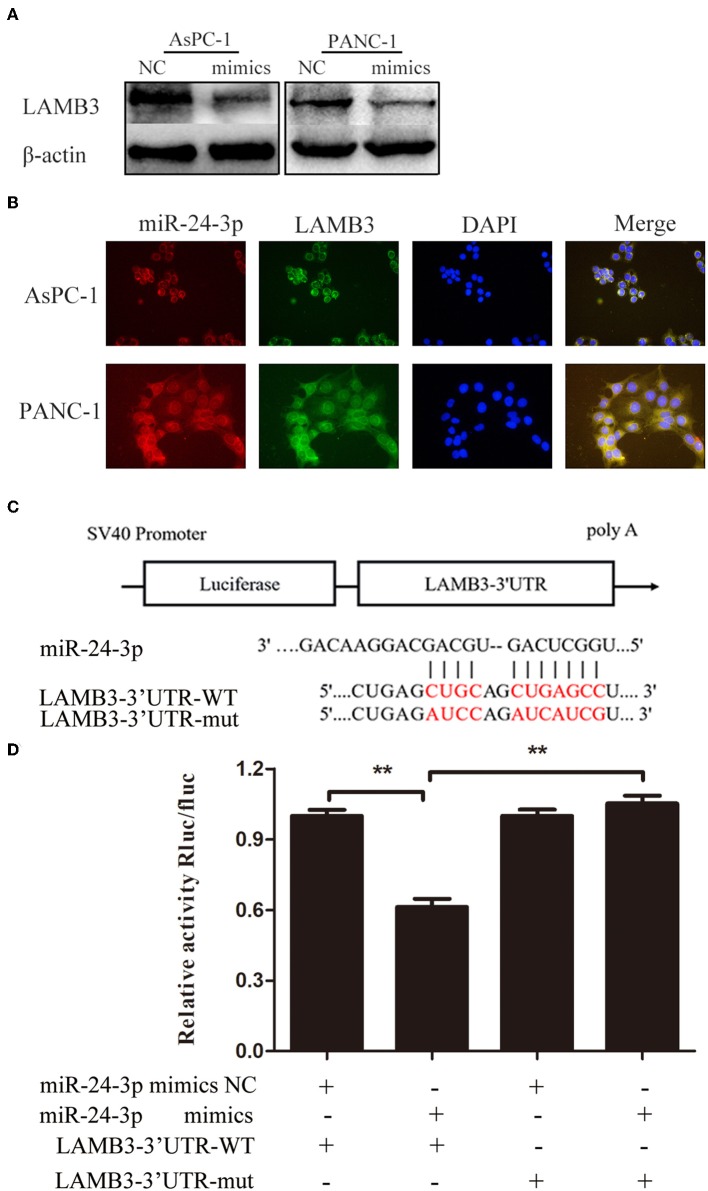
MiR-24-3p targets LAMB3 directly. **(A)** Western blotting analysis showed that LAMB3 expression was reduced after transfection with miR-24-3p mimics. **(B)** FISH assay demonstrated that miR-24-3p and LAMB3 mRNA were predominantly localized in the cytoplasm, and co-localization of miR-24-3p and LAMB3 mRNA was observed in PDAC cells. **(C)** A schematic showing the putative binding sites of miR-24-3p with respect to the 3'UTR of LAMB3. **(D)** Luciferase assay confirmed that miR-24-3p targeted the 3′UTR region of LAMB3. The data are presented as mean ± SD of three independent experiments. **p < 0.01, Student's *t*-test.

As a previous study demonstrated that most mammalian mRNAs could serve as conserved targets of miRNAs, we investigated whether LAMB3 was regulated by miRNAs (25). Using several databases, including PITA, miRmaP, miRanda, and Targetscan, we found that miR-24-3p was one of the potential upstream regulators of LAMB3, which was supported by two AGO CLIP-seq experiments (26). To explore whether miR-24-3p regulated LAMB3 expression, miR-24-3p mimics were introduced into AsPC-1 and PANC-1 cells. Western blotting revealed that ectopic miR-24-3p overexpression inhibited LAMB3 expression (Figure 3A). The subcellular location of miR-24-3p and LAMB3 was investigated. By using FISH analysis, we found that miR-24-3p was co-localized with LAMB3 in the cytoplasm (Figure 3B). A dual-luciferase reporter system was adopted to validate whether miR-24-3p targeted the LAMB3 3'UTR directly. In this section, miR-24-3p mimics, plasmids containing wild or mutant types of the seed region in the LAMB3 3'UTR, were constructed (Figure 3C). Co-transfection of miR-24-3p mimics with wild type plasmid significantly reduced the luciferase activity compared to with mutant type plasmid (Figure 3D). We next investigated the expression of miR-24-3p in 15 matching pancreatic cancer and adjacent non-cancerous tissues. qRT-PCR showed that miR-24-3p was significantly downregulated in PDAC tissues (Supplementary Figure 1A). To investigate whether miR-24-3p was associated with the clinical prognosis of PDAC patients, we examined the miR-24-3p expression level in 50 PDAC tissues. miR-24-3p expression in PDAC was divided into two groups: the high group (*n* = 31) and the low group (*n* = 29). Kaplan-Meier survival curve analysis revealed that patients with high miR-24-3p expression had a higher survival rate compared to those with low miR-24-3p expression (log-rank test, *p* < 0.001, Supplementary Figure 1B).

In conclusion, miR-24-3p is downregulated in PDAC tissues, and reduced miR-24-3p expression may serve as an indicator of poor prognosis. Additionally, miR-24-3p inhibits LAMB3 expression by directly targeting the 3′UTR of LAMB3 mRNA.

### Overexpression of LAMB3 Reverses miR-24-3p Mimics-Induced Inhibition of Cell Proliferation, the Cell Cycle, and Invasion

**Figure 4:**
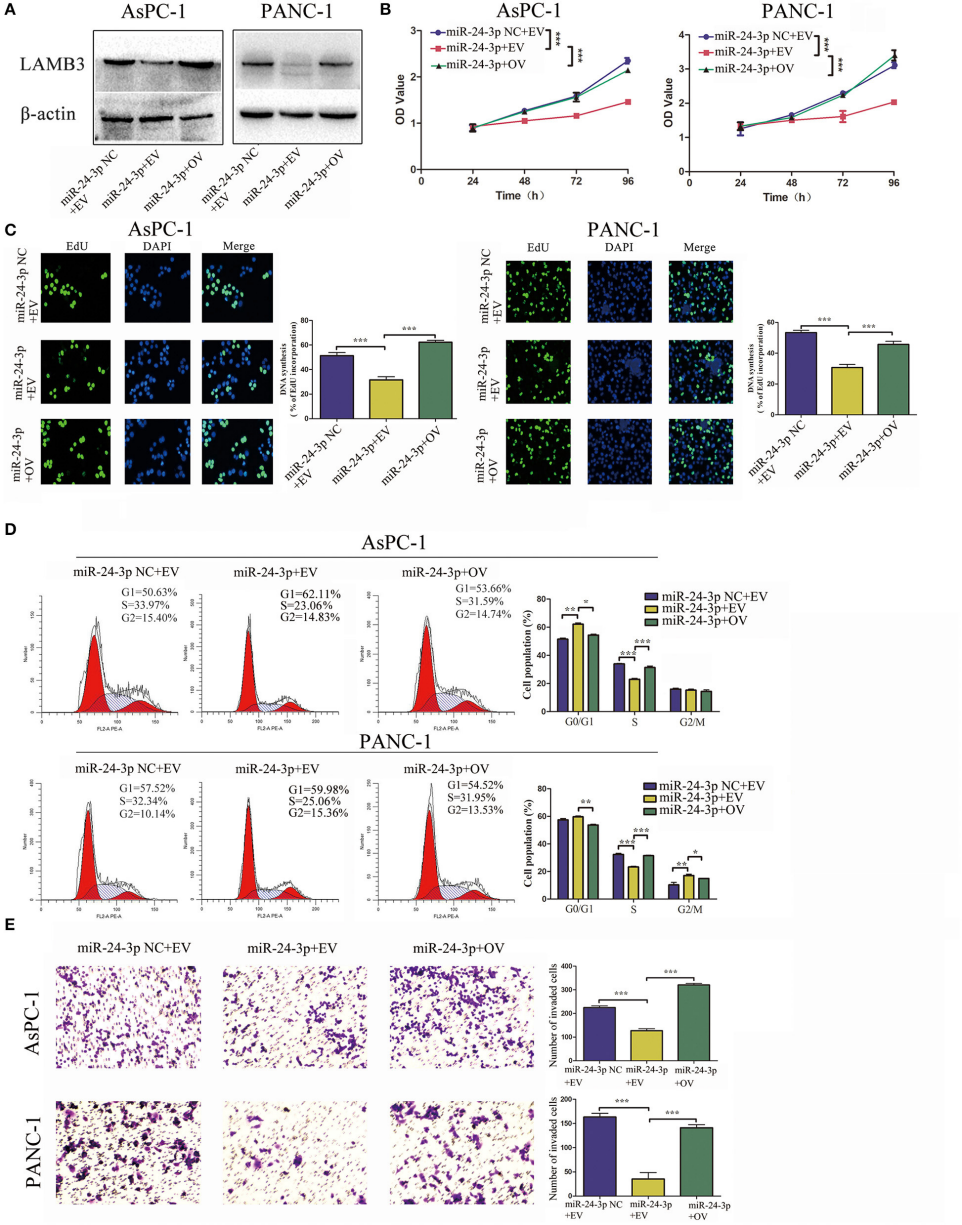
MiR-24-3p overexpression inhibits tumor progression, which is recovered by the reintroduction of LAMB3. **(A)** LAMB3 expression levels were determined using Western blotting in cells transfected with miR-24-3p mimics and LAMB3 overexpression vector (OV). Cells transfected with LAMB3 empty vector (EV) and mimics NC were used as the negative controls. **(B,C)** CCK-8 and EdU assays showed that miR-24-3p overexpression inhibited cell proliferation, whereas overexpression of LAMB3 rescued the inhibitory effects. **(D)** Flow cytometry revealed that miR-24-3p overexpression induced cell cycle arrest, whereas overexpression of LAMB3 recovered the cell cycle period. **(E)** Transwell assays confirmed that miR-24-3p overexpression impaired the cell invasion capacity. Overexpression of LAMB3 resulted in enhanced cell invasion capacity. Data are presented as mean ± SD of three independent experiments; *p < 0.05, **p < 0.01, ***p < 0.001, paired *t*-test.

We further investigated whether miR-24-3p exerted its function via LAMB3. miR-24-3p mimics were transfected into PDAC cells to ectopically upregulate miR-24-3p. To investigate the direct antagonizing effect of miR-24-3p, LAMB3 OV without the 3′UTR was reintroduced into miR-24-3p-overexpressing cells. Cells transfected with miR-24-3p mimics NC and LAMB3 empty vector served as negative controls. Western blotting revealed that miR-24-3p overexpression inhibited LAMB3 expression and that the reintroduction of LAMB3 restored its expression (Figure 4A). CCK-8 and EdU assays revealed that the proliferation rate was significantly inhibited in miR-24-3p-overexpressing cells. Re-expression of LAMB3 could rescue the inhibitory effects (Figures 4B,C). Flow cytometry revealed that miR-24-3p overexpression induced cell cycle arrest, whereas the reintroduction of LAMB3 abrogated this effect (Figure 4D). Transwell invasion assay confirmed that miR-24-3p overexpression impaired the metastatic properties and that regaining LAMB3 expression restored the invasion capacity (Figure 4E).

### Silencing of LAMB3 Abrogates miR-24-3p Inhibitor-Induced Acceleration of Cell Proliferation, the Cell Cycle, and Invasion

**Figure 5:**
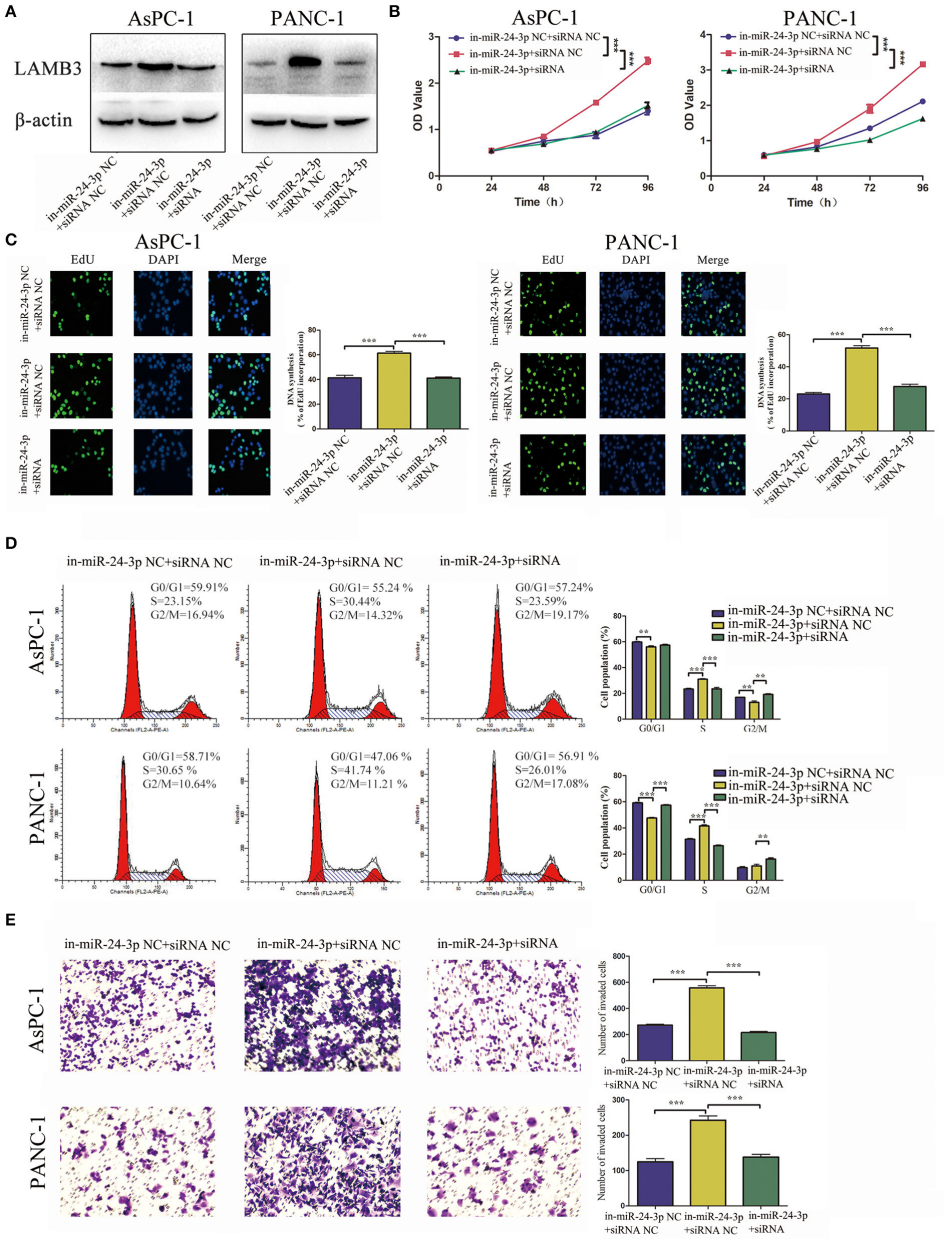
MiR-24-3p inhibition promotes tumor progression and is rescued by co-transfection of LAMB3 siRNA. **(A)** LAMB3 expression levels were determined using Western blotting analysis in cells transfected with miR-24-3p inhibitor and LAMB3 siRNA NC (in-miR-24-3p + siRNA NC) and miR-24-3p inhibitor and LAMB3 siRNA (in-miR-24-3p + siRNA). Cells transfected with LAMB3 siRNA NC and inhibitor NC were used as the negative controls. **(B,C)** CCK-8 and EdU assays showed that miR-24-3p inhibition promoted cell proliferation, whereas LAMB3 knockdown abrogated these effects. **(D)** Flow cytometry revealed that miR-24-3p inhibition accelerated the cell cycle, whereas knockdown of LAMB3 induced cell cycle arrest. **(E)** Transwell assays revealed that miR-24-3p inhibition enhanced the cell invasion capacity, whereas LAMB3 knockdown impaired the cell invasion capacity. Data are presented as mean ± SD of three independent experiments; **p < 0.01, ***p < 0.001, paired *t*-test.

For reversed verification, miR-24-3p inhibitor was transfected into PDAC cells to inhibit miR-24-3p, and LAMB3 siRNA was reintroduced into miR-24-3p knockdown cells. Cells transfected with miR-24-3p inhibitor NC and LAMB3 siRNA NC were used as negative controls. Western blotting revealed that miR-24-3p knockdown promoted LAMB3 expression and that co-transfection with LAMB3 siRNA inhibited the LAMB3 expression (Figure 5A). CCK-8 and EdU assays revealed that cells transfected with miR-24-3p inhibitor showed elevated proliferative capacities. However, co-transfection of LAMB3 abrogated this effect (Figures 5B,C). Flow cytometry further revealed that miR-24-3p knockdown accelerated the cell cycle and that co-transfection of LAMB3 siRNA induced cell cycle arrest (Figure 5D). Transwell assays revealed that miR-24-3p knockdown enhanced the invasion capacity, and the rescue assay showed that co-transfection of LAMB3 suppressed the invasion capacity in both AsPC-1 and PANC-1 cell lines (Figure 5E).

Since overexpression of LAMB3 abrogated the anti-cancer effects induced by miR-24-3p mimics and silencing of LAMB3 abrogated the oncogenic effects induced by miR-24-3p inhibitor, we concluded that miR-24-3p might exert anti-tumor effects by regulating LAMB3 expression *in vitro*.

### MiR-24-3p Inhibits Tumor Growth *in vivo*

**Figure 6:**
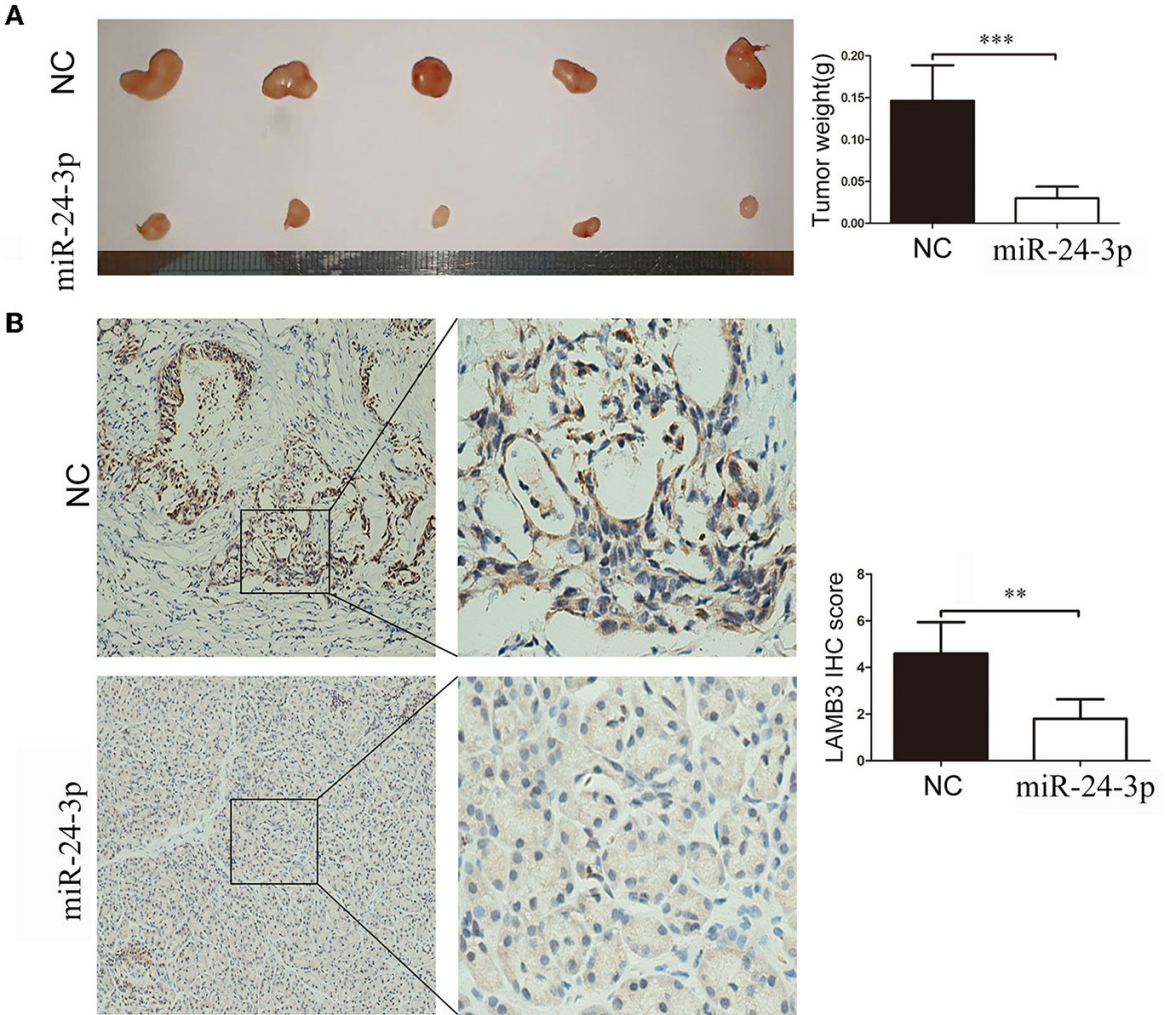
MiR-24-3p inhibits pancreatic cancer proliferation *in vivo*. **(A)** In total, 2 × 10^6^ AsPC-1 cells were implanted subcutaneously in nude mice, and the tumor weight was evaluated after 20 days. **(B)** IHC results showed that miR-24-3p mimics inhibited LAMB3 expression. Data are presented as mean ± SD of three independent experiments; **p < 0.01, ***p < 0.001, paired *t*-test.

We validated the anti-cancer effects of miR-24-3p *in vivo*. AsPC-1 cells were transfected with miR-24-3p mimics or NC and implanted into the backs of nude mice. After 20 days, the mice were sacrificed, and the weight of the tumor was recorded. As shown in Figure 6A, tumors derived from cells transfected with miR-24-3p mimics had a lower weight than those transfected with NC. We examined the LAMB3 expression in the two groups. IHC results showed that the miR-24-3p mimics group had lower LAMB3 expression compared to the NC group (Figure 6B). Our *in vivo* experiments confirmed that miR-24-3p repressed tumor proliferation and inhibited the expression of LAMB3.

## Discussion

Previous studies have demonstrated the complex and even paradoxical roles of miR-24-3p in various types of cancers. In lung cancer (LC), the overexpression of miR-24-3p promoted cell proliferation and migration by suppressing SOX7 (27). In addition, its oncogenic role was also reported in hepatocellular carcinoma (28) and nasopharyngeal carcinoma (29). In addition, tumor-suppressing roles of miR-24-3p have been reported in lung adenocarcinoma cell lines and colorectal cancer (CRC) (30, 31). However, no studies investigating the expression profiles and biological roles of miR-24-3p in pancreatic cancer have yet been reported. In our study, clinicopathological feature analysis showed that miR-24-3p was downregulated in PDAC tissues compared to adjacent non-cancer tissues and that low expression of miR-24-3p was associated with poorer overall survival of PDAC patients. Functional studies *in vitro* revealed that miR-24-3p served as a tumor suppressor in PDAC progression. Using bioinformatics analysis, we predicted that miR-24-3p was a potential upstream miRNA of LAMB3. We further investigated the underlying mechanisms of the miR-24-3p/LAMB3 axis in PDAC.

The important roles of LAMB3 in cancer are well-known. In thyroid cancer and head and neck squamous cell carcinoma (HNSCC), LAMB3 is significantly upregulated and promotes proliferation and metastasis (20, 32). In pancreatic cancer, LAMB3 plays important roles in cell cycle arrest, apoptosis, proliferation, and invasion by regulating the PI3K/Akt signaling pathway (33). Despite its oncogenic role, LAMB3 can also act as a tumor suppressor gene in hepatocellular carcinoma progression (34). Previous studies have reported that LAMB3 is also targeted by some miRNAs, such as miR-1298 and miR-329, regulating cancer progression (35, 36). In this study, we illustrated that LAMB3 was upregulated in PDAC tissues compared to non-cancerous tissues and was significantly associated with PDAC prognosis. Functional studies confirmed that LAMB3 served as an oncogene in PDAC progression. The above-stated results indicate that LAMB3 may serve as a potential prognostic and therapeutic biomarker in PDAC.

Using dual-luciferase reporter assay and FISH assay, we confirmed that miR-24-3p inhibited LAMB3 expression by directly targeting the 3'UTR of LAMB3 at the post-transcriptional level, suggesting that the absence of miR-24-3p was one putative mechanism responsible for the aberrant overexpression of LAMB3. Functional studies confirmed that regaining LAMB3 in miR-24-3p-overexpressing cells abrogated the anti-cancer effect induced by miR-24-3p and that LAMB3 inhibition abrogated the cancer-promoting effects induced by the miR-24-3p inhibitor. Thus, we demonstrated that miR-24-3p-regulated LAMB3 contributed to the proliferation, cell cycle, and invasion of PDAC *in vitro*. Accumulating evidence has confirmed that the ectopic expression of miRNA by mimics shows an anti-tumor property and may serve as a promising therapeutic strategy (37). We also confirmed that the restoration of miR-24-3p inhibited PDAC growth and LAMB3 expression in a subcutaneous xenotransplanted tumor model *in vivo*.

In conclusion, our results indicate that LAMB3 is upregulated in PDAC and may serve as an oncogene in PDAC. miR-24-3p is a novel regulatory factor of LAMB3 and exerts anti-tumor effects by suppressing LAMB3 expression *in vitro* and *in vivo*. The miR-24-3p/LAMB3 axis plays a vital role in tumor progression, and targeting it may represent a novel therapeutic strategy for PDAC.

## Materials and Methods

### Clinical Samples and Cell Lines

Fifteen pairs of fresh primary PDAC and adjacent non-cancerous tissues and fifty PDAC tissues were used in this study. The tissues were preserved in liquid nitrogen after surgical resection at Southern Medical Hospital. All patients provided signed informed consent. Two PDAC cell lines (AsPC-1 and PANC-1) used in this study were obtained from Procell Life Science & Technology Co., Ltd. (Wuhan, China). All cells were cultured in DMEM with 10% fetal bovine serum (FBS) and 1% Penicillin Streptomycin in a humidified 5% CO_2_ atmosphere at 37°C.

### GEO and TCGA Data Collection and Analysis

We searched the GEO database to identify the altered gene expression in PDAC and adjacent non-cancerous tissues. In the GSE28735 dataset, exploring the gene expression profiles of 45 matched pairs of PDAC and adjacent non-tumor tissues from 45 patients using a microarray, we found that LAMB3 was aberrantly upregulated. The GSE16515 and GSE62452 datasets confirmed our findings. To investigate the correlation of LAMB3 and PDAC prognosis in the TCGA database, 176 PDAC cases that recorded LAMB3 expression profiles along with detailed clinical information were downloaded from cBioPortal.org.

### RNA Extraction

TRIzol regent (Invitrogen, Camarillo, CA, USA) was used for RNA extraction according to the manufacturer's instruction, and 1 mL TRIzol was used per 30 mg of PDAC tissue or 10^6^ PDAC cells. After extraction, RNA concentration was determined, and RNA was stored at −80°C to avoid degradation.

### Cell Transfection

MiR-24-3p mimics, mimics NC, miR-24-3p inhibitor, inhibitor NC, LAMB3 siRNA, and LAMB3 siRNA NC were purchased from RiboBio (Guangzhou, China). The LAMB3 overexpression vector was purchased from GeneChem (Shanghai, China). Transfection was performed using a Lipofectamine 3000 (Invitrogen).

### qRT-PCR (Quantitative Reverse Transcription PCR)

For LAMB3, qRT-PCR analysis was performed using a PrimeScript RT reagent kit (Takara) and an SYBR Premix Ex Taq II (Takara). β-actin was used as an endogenous control. For miR-24-3p, a Mir-X miR First-Strand Synthesis kit (Takara) and an SYBR Premix Ex Taq II (Takara) were used for qRT-PCR. RNU6-2 was used as an endogenous control. The 2^−Δ*ΔCT*^ method was used to calculate the relative expressions of miR-24-3p and LAMB3. RiboBio designed the bulge-loop miRNA qRT-PCR primer sets specific for miR-24-3p. Sangon Biotech synthesized the LAMB3 primer with sequences 5′-AGCTTTCAGGCGATCTGGAG-3′ (forward) and 5′-GTCTCAGGCTTGGTCAGTCC-3′ (reverse).

### Protein Extraction and Western Blotting

Protein lysates were extracted using RIPA (Beyotime Institute of Biotechnology, Shanghai, China) as per the manufacturer's instructions. After quantification, protein lysates were separated on SDS-PAGE gel and transferred onto polyvinylidene fluoride membranes (Millipore, Billerica, MA). Membranes were blocked with 5% skim milk for 1 h at room temperature and incubated with primary antibody at 4°C overnight. After washing with TBS-T, membranes were incubated with horseradish peroxidase (HRP)-conjugated secondary antibody at room temperature for 2 h. β-actin served as a loading control. LAMB3, β-actin antibodies, and secondary antibody were purchased from Proteintech (Wuhan, China). The original image files of the Western Blotting was presented in Supplementary Data Sheet 1.

### Cell Proliferation Assays

The proliferative capacity of PDAC cells was determined using the Cell Counting Kit-8 (CCK-8) assay and the 5-Ethynyl-2′-deoxyuridine (EdU) incorporation assay. CCK-8 assay was performed as per the manufacturer's instructions (Dojindo, Shanghai, China). In this study, 5,000 cells were seeded into 96-well-plates, and 10 μL CCK-8 solution was added to each well. The OD value was recorded at 24, 48, 72, and 96 h after transfection. The EdU assay was performed as per the manufacturer's instructions (RiboBio).

### Cell Cycle

Flow cytometry was used for cell cycle analysis. After experimental treatment, no <10^5^ cells were collected, fixed in 70% ethanol overnight at −20°C, and stained with propidium iodide (Kaiji, Nanjing, China) in a phosphate-buffered saline solution containing RNase. The fixed cells were analyzed using flow cytometry (Beckman FC500, Los Angeles, CA, USA).

### Luciferase Reporter Assay

The wild and mutant types of the 3'UTR sequence of LAMB3 were inserted into the siCheck2 vector at a position downstream of the SV40 promoter. Mutations were generated in the binding sites of the 3′UTR. Next, 0.16 μg plasmid containing the LAMB3-3′UTR/LAMB3-3′UTR mutant and 5 pmol hsa-miR-24-3p/Negative Control (NC) were transfected into 293T cells independently. After 48 h, Firefly luciferase (internal reference) and Renilla luciferase activities were measured using the Promega Dual-Luciferase system according to the manufacturer's protocol.

### Invasion Assay

Invasion assay was performed using Matrigel Invasion Chambers (Merck Millipore, USA) according to the manufacturer's instructions. After 24-h transfection, 20,000 cells suspended in serum-free DMEM were seeded onto the upper chambers (8-μm pore size), and DMEM with 10% FBS was added to the lower chambers. After 48 h, cells that migrated to the bottom of the insert membrane were fixed with 4% paraformaldehyde and stained with crystal violet.

### Fluorescence *in situ* Hybridization (FISH)

Hybridization was performed overnight with hsa-miR-24-3p and LAMB3 probes. Specimens were analyzed on a Nikon inverted fluorescence microscope. The hsa-miR-24-3p probe for FISH was 5′-CTGTTCCTGCTGAACTGAGCCA-3′, and the LAMB3 probe for FISH was 5′-CCCTGCGGGTTGGC GTAGGTGAGT-3′.

### Immunohistochemistry (IHC)

Immunohistochemistry was performed following the manufacturer's instructions (Maixin, Fuzhou, China). Briefly, after antigen retrieval, the sections were incubated overnight with primary antibodies against LAMB3 at 4°C. They were incubated with secondary antibodies, followed by incubation with the streptavidin-biotin complex (Maixin). Staining intensity was scored as negative staining (score = 0), weak staining (score = 1), moderate staining (score = 2), or strong staining (score = 3). The staining extent was based on the percentage of positive cells (0, 0%; 1, <10%; 2, 10–50%; 3, > 50%). Immunoreactivity was quantified by multiplying the staining extent score by the intensity score.

### Animal Experiments

Four-week-old female nude mice were purchased from the Peking University Animal Center (Beijing, China). After 5 days of acclimatization, a total of 2 × 10^6^ AsPC-1 cells transfected with either miR-24-3p or NC were injected subcutaneously into the right back of each mouse. The mice were killed, and tumor weights were measured and recorded in grams on the 20th day after injection. All animal studies were approved by the Institutional Animal Care and Use Committee of the Army Medical University.

### Statistical Analysis

Statistical analysis was performed using GraphPad Prism 7 software. Quantitative data were presented as mean ± standard deviation (SD). The paired two-tailed Student's *t*-test or unpaired *t*-test was used for quantitative data, as appropriate. Kaplan-Meier analysis with log-rank testing was applied for survival analysis. Differences were considered statistically significant when *p* < 0.05 (^*^p < 0.05; ^**^p < 0.01; ^***^p < 0.001).

## Data Availability Statement

Publicly available datasets were analyzed in this study. This data can be found here: http://www.proteinatlas.org.

## Ethics Statement

The studies involving human participants were reviewed and approved by Department of Hepatobiliary Surgery, Zhujiang Hospital, Southern Medical University, Guangzhou, Guangdong, China. Written informed consent for participation was not required for this study in accordance with the national legislation and the institutional requirements. The animal study was reviewed and approved by Department of Hepatobiliary Surgery, Zhujiang Hospital, Southern Medical University, Guangzhou, Guangdong, China.

## Author Contributions

HW and YF: study concept and design, critical revision of the manuscript for important intellectual content, and technical support. WH: study concept and design, acquisition of data, analysis and interpretation of data, obtaining the findings, and drafting of the manuscript. JG and JZ: acquisition of data. TT: material support.

### Conflict of Interest

The authors declare that the research was conducted in the absence of any commercial or financial relationships that could be construed as a potential conflict of interest.

## Supplementary Material

Supplementary Figure 1 miR-24-3p is downregulated in PDAC tissues and is associated with the prognosis of PDAC patients. **(A)** Lower expression levels of miR-24-3p were detected using qRT-PCR in 15 PDAC tissues compared to the adjacent non-tumor tissues. Data are presented as mean ± SD of three independent experiments; ^***^*p* < 0.001, paired *t*-test. **(B)** Kaplan-Meier analysis revealed that low expression of miR-24-3p was associated with poorer overall survival of PDAC patients. *p* < 0.001, log-rank test.

## Abbreviations

PDAC: pancreatic ductal adenocarcinoma
LAMB3: laminin subunit beta 3
DMEM: Dulbecco's modified Eagle's medium
FBS: fetal bovine serum
TCGA: The Cancer Genome Atlas
GEO: Gene Expression Omnibus
qRT-PCR: quantitative reverse transcription PCR.
