# Supplementary figures and images for: MiR-24-3p Inhibits the Progression of Pancreatic Ductal Adenocarcinoma Through LAMB3 Downregulation
Source: Front Oncol. 2020 Jan 21;9:1499. doi: 10.3389/fonc.2019.01499 (PMC6985431; doi:10.3389/fonc.2019.01499)

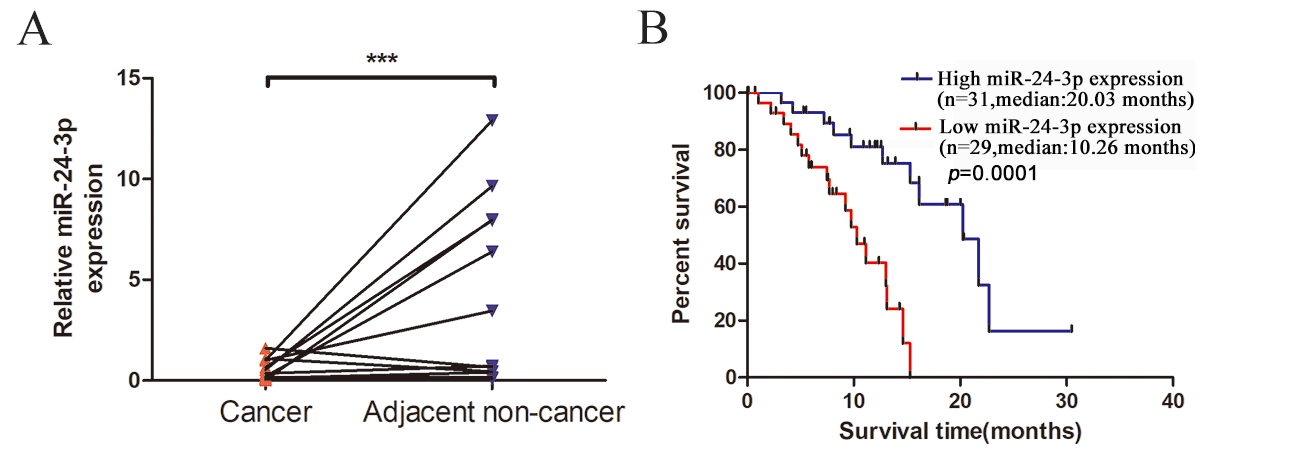

Supplement: Supplementary Figure 1 — miR-24-3p is downregulated in PDAC tissues and is associated with the prognosis of PDAC patients. (A) Lower expression levels of miR-24-3p were detected using qRT-PCR in 15 PDAC tissues compared to the adjacent non-tumor tissues. Data are presented as mean ± SD of three independent experiments; ***p < 0.001, paired t-test. (B) Kaplan-Meier analysis revealed that low expression of miR-24-3p was associated with poorer overall survival of PDAC patients. p < 0.001, log-rank test. [file Image_1.tif]
