# Supplementary material for: MiR-24-3p Inhibits the Progression of Pancreatic Ductal Adenocarcinoma Through LAMB3 Downregulation
Source: Front Oncol. 2020 Jan 21;9:1499. doi: 10.3389/fonc.2019.01499 (PMC6985431; doi:10.3389/fonc.2019.01499)
Supplement: Supplementary file 2 [file Data_Sheet_1.docx]

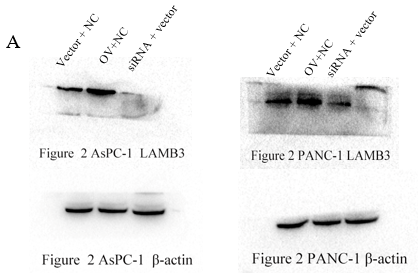

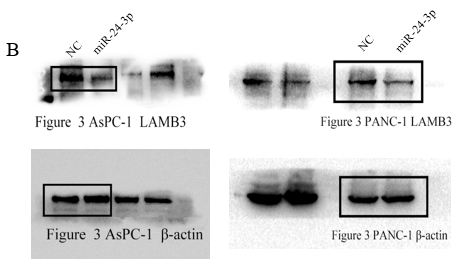


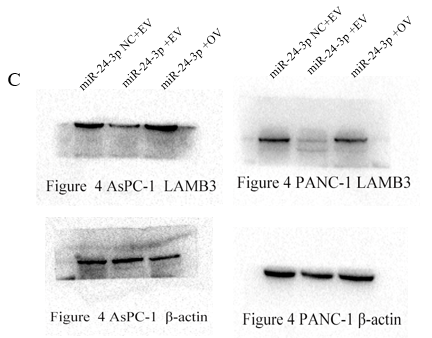


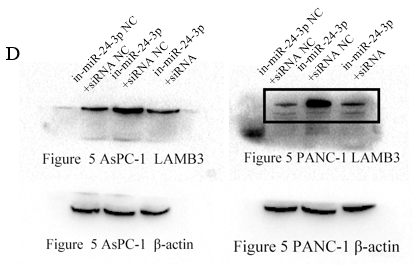


**Data sheet 1. (A) The original image files for the blots of figure 2 .LAMB3 expression levels were determined using western blotting analysis in cells transfected with LAMB3 overexpression vector (OV), LAMB3 siRNA. Cells transfected with LAMB3 empty vector (EV) and siRNA NC were used as the negative controls. (B) The original image files for the blots of figure 3.** **Western blotting analysis showed that LAMB3 expression was reduced after transfection with miR-24-3p mimics. Cells transfected with miR-24-3p mimics NC were used as the negative controls. (C) The original image files for the blots of figure 4.** **LAMB3 expression levels were determined using western blotting in cells transfected with miR-24-3p mimics, and LAMB3 overexpression vector (OV). Cells transfected with LAMB3 empty vector (EV) and mimics NC were used as the negative controls. (D) The original image files for the blots of figure 5.** **LAMB3 expression levels were determined using western blotting analysis in cells transfected with miR-24-3p inhibitor and LAMB3 siRNA NC (in-miR-24-3p + siRNA NC) and miR-24-3p inhibitor and LAMB3 siRNA (in-miR-24-3p + siRNA). Cells transfected with LAMB3 siRNA NC and inhibitor NC were used as the negative controls.**
